# Misunderstandings Between Platelets and Neutrophils Build in Chronic Inflammation

**DOI:** 10.3389/fimmu.2019.02491

**Published:** 2019-10-22

**Authors:** Giuseppe A. Ramirez, Angelo A. Manfredi, Norma Maugeri

**Affiliations:** ^1^Vita-Salute San Raffaele University, Milan, Italy; ^2^Division of Immunology, Transplantation and Infectious Diseases, IRCCS Ospedale San Raffaele, Milan, Italy

**Keywords:** platelets, neutrophil, inflammation, autoimmunity, systemic lupus erythematosus, systemic sclerosis, rheumatoid arthritis, vasculitis

## Abstract

Regulated hemostasis, inflammation and innate immunity entail extensive interactions between platelets and neutrophils. Under physiological conditions, vascular inflammation offers a template for the establishment of effective intravascular immunity, with platelets providing neutrophils with an array of signals that increase their activation threshold, thus limiting collateral damage to tissues and promoting termination of the inflammatory response. By contrast, persistent systemic inflammation as observed in immune-mediated diseases, such as systemic vasculitides, systemic sclerosis, systemic lupus erythematosus or rheumatoid arthritis is characterized by platelet and neutrophil reciprocal activation, which ultimately culminates in the generation of thrombo-inflammatory lesions, fostering vascular injury and organ damage. Here, we discuss recent evidence regarding the multifaceted aspects of platelet-neutrophil interactions from bone marrow precursors to shed microparticles. Moreover, we analyse shared and disease-specific events due to an aberrant deployment of these interactions in human diseases. To restore communications between the pillars of the immune-hemostatic continuum constitutes a fascinating challenge for the near future.

## Introduction

### The Immune-Hemostatic Continuum

The circulatory system provides functional integration to tissues throughout the body and constitutes a dynamic platform for tasks, such as immune patrolling and defense against threats (1–3). Consistently, abnormalities in blood cells and cardiovascular manifestations are disproportionately represented in systemic autoimmune diseases, reflecting a network of interactions among circulating elements in the blood (4–7). Humoral moieties, such as complement, opsonins, components of the coagulation cascade and regulators of the vascular tone lie at the lowest level of complexity in this system and provide immediate, stereotyped responses to abnormal changes in the environment, such as volume loss, vascular injury or pathogen invasion, besides supporting more elaborate, long-term tasks performed by cells or subcellular elements (8–11).

Cellular membranes allow the segregation of selected information in compartments and modulate their subsequent effects on the environment by integrating multiple stimuli. Circulating membrane-endowed players in the immuno-hemostatic network encompass leukocytes, platelets and microparticles, with the endothelium as a fourth static counterpart (12, 13). Platelets and neutrophils play a crucial role in the maintenance of vascular and tissue integrity and interact extensively and productively. Consistently, alterations in platelet-neutrophil cross-talk have dramatic long-range effects on vascular and immune homeostasis (14–16) and are growingly appreciated as targets for therapeutic intervention (17, 18).

## Pathophysiology of Interactions Between Platelets and Neutrophils

### Characters on the Stage: Platelets, Neutrophils, and Microparticles

Hemostasis and inflammation counterbalance the effect of injuring external stimuli. Selective regulation and polarization of these pathways enhance homeostatic responses at sites of tissue or vascular injury and minimize the detrimental effects to the host. Multiple mechanisms have developed, including variability in the lifespan of players involved in the immune-hemostatic balance and availability of soluble or membrane-bound moieties. Emission of membrane-endowed subcellular particles fine-tunes cellular activation and converts locally concentrated high-intensity responses into a sum of smaller but widespread and reciprocally independent biological events. Generation of extracellular vesicles enables the extension of the total membrane area interacting with the environment as well as of the range of potential cellular targets. In addition, segregation of information in multiple signaling quanta discloses the possibility of independent interactions with distinct cellular counterparts according to the differential needs of target tissues. Platelets are anucleate cell fragments released by megakaryocytes. After a multi-stage process of cytoplasm compartmentalisation and concentration of bioactive compounds into granules taking place over the course of days, platelets are released as elongated precursors (proplatelets), which undergo multiple iterative fission events to reach their final size. Preplatelets are round-shaped precursors constituting a reversible intermediate stage in the transition from proplatelets to platelets (19). Small vessels of the bone marrow, spleen and lung might deliver signals that facilitate the final process of platelet maturation (20).

Increased platelet demand and/or consumption during acute systemic inflammation warrants adaptation of megakaryocytes. Inflammatory cytokines, such as IL6 promote megakaryocyte increase in ploidy and prompt thrombocytopoiesis through increased liver synthesis of thrombopoietin as part of the acute phase response (21). Platelets released under inflammatory stress are usually larger in volume, which correlates with an increased ischemic risk at a clinical level (22). Alternative sites of thrombocytopoiesis, such as the lung, might become activated under stress conditions in mice (23) and possibly in patients with lung cancer (24). In addition, stem-like megakaryocyte progenitors can be activated on demand during interferon-α-driven inflammatory responses (25). Platelets survive for 7–10 days in circulation, where they surrogate the damaged endothelium during vascular injury, recognize and control invading pathogens and release stimuli to promote tissue repair (12).

Controlled exocytosis or integration of bioactive compounds into platelet membrane is crucial for these tasks. Platelets are endowed with three classes of granules: alpha-granules; dense granules and few lysosomes. Some authors also described “T granules” equipped with Toll-like receptor 9 as a potential fourth platelet compartment (26–28). Besides being providers of bioactive compounds through exocytosis, platelets also produce microparticles. Platelet-derived microparticles (PDμP) constitute a substantial fraction of circulating microparticles in humans under physiological conditions (29). PDμP can present with a variety of sizes, contents and functions (30–32) that range from facilitation of coagulation through tissue factor (TF) and phospholipid (phosphatidylserine) scaffolds (33, 34) to angiogenesis, tissue repair (35–37) and defensive responses (38). Modulation of neutrophil behavior through delivery of nucleic acids (RNA) or inflammatory signal intercellular transfer also occurs under inflammatory conditions (39–42). In addition, mitochondria-enriched PDμP modulate target cell metabolism (32). Megakaryocytes release microparticles as well, influencing bone marrow homeostasis and synergising with PDμP (32, 43, 44).

Neutrophils constitute the most abundant leukocyte population in the blood and are in charge of the early innate effector response to noxious stimuli (45). Neutrophils express a vast array of oxidative and proteolytic enzymes, which are preformed and stored in ready-to-use granules. Activated neutrophils express TF, promoting isolation of injured tissues through thrombosis (46, 47) and contributing to immune-thrombosis upon interaction with platelets (2, 3). Neutrophils have a limited lifespan (45), which is mirrored by the timing of multiple acute or hyperacute clinical manifestations of infectious and immune-mediated diseases (48–50). Autophagy induction under inflammatory conditions might extend neutrophil survival, causing chronicization of tissue damage and facilitating autoimmunity (40, 51–55). In addition hematopoietic stem cells and myeloid progenitors respond to extreme inflammatory stimuli (thanks to the expression of innate germ-line encoded receptors, such as Toll-like receptors) causing massive granulopoiesis (56, 57).

Phagocytosis and digestion of invading pathogens constitutes the default-mode defensive task performed by neutrophils. However, frustrated microbial phagocytosis promotes the generation of extracellular traps (NETs) (58), i.e., the extracellular release of threads of decondensed chromatin and microbicidal moieties with or without loss of membrane integrity and cell vitality (suicidal vs. vital NET generation) (59). At least in cases leading to cell death, granule content is partially repurposed to facilitate chromatin remodeling and histone citrullination (60–63). Besides having a role in antimicrobial defense, NETs are also generated in response to unconventional stimuli, such as amorphous crystals, apoptotic bodies, cytokines, microparticles and changes in osmolarity (64, 65). Factors driving neutrophils to “choose” NET generation as opposed to phagocytosis are partially understood (66). Unsolicited formation and impaired clearance of NETs implies persistent exposure of self-antigens and inflammatory stimuli, which facilitates the development of autoimmunity (67–73).

Neutrophils also account for tissue and vascular damage in immune-mediated diseases including inflammatory bowel diseases, systemic lupus erythematosus (SLE) rheumatoid arthritis (RA) and vasculitides either directly or through NET-induced facilitation of thrombosis (2, 67, 68, 74–76). Neutrophils cooperate to regulate immune activation by secreting soluble pattern recognition receptors, such as pentraxin-3 (PTX3) or ficolins (3, 77, 78). Furthermore, they influence the activation and survival of other immunocompetent cells and tune the degree of systemic inflammation (45). Finally, neutrophils emit microparticles loaded with nucleic acids and/or digestive enzymes and armed with tissue factor (47, 79, 80). Neutrophil-derived microparticles prime endothelial cells, macrophages and neutrophils themselves to inflammatory activation (80, 81) and possibly destabilize genomic integrity in target tissues preventing resolution of the immune response (82). Elevated levels of such microparticles, possibly interacting with NET constituents are detectable in blood of patients with immune-mediated diseases (83, 84).

### Weaving the Plot: General Features of Platelet-Neutrophil Interactions

**Figure 1:**
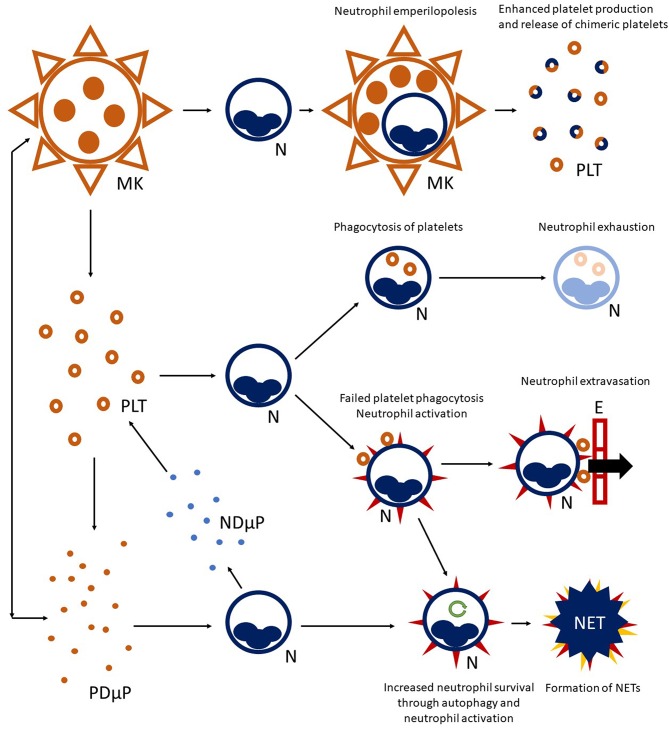
Platelet-neutrophil crosstalk. A complex network of interactions connects platelet (PLT) to neutrophil (N) biology. Megakaryocytes (MK) are able to interact with neutrophils residing in the bone marrow and engulf them, preserving their vitality (a phenomenon called emperilopolesis). Neutrophils may eventually escape megakaryocyte engulfment after donating membrane components to the host cell. This event causes enhanced platelet production and release of chimeric platelets. Activated platelets and neutrophils can further interact in the circulating blood, either directly through cell-cell contact and/or through the exchange of soluble compounds or microparticles. Engagement of platelets by neutrophils through the P-selectin/PSGL-1 axis, and later on, integrin-mediated bonds can lead to platelet-phagocytosis, resulting in neutrophil exhaustion. Alternatively, failed platelet clearance can promote neutrophil activation (heralded by expression of surface markers, such as tissue factor and activation of integrins—red spikes in the figure) and facilitate neutrophil extravasation through the endothelial wall (E). Activated neutrophils can also enter autophagy programmes (green circular arrow), extending their survival, and progress toward the formation of neutrophil extracellular traps (NET). Platelet-derived microparticles (PDμP), especially when loaded with the damage associated molecular pattern HMGB1, are potent inducers of NET generation. Neutrophil-derived microparticles (NDμP) constitute and additional channel for platelet-neutrophil interchanges and are thought to have a role in lipid metabolism.

Platelets interact productively with multiple cells types (12), although neutrophils constitute preferential partners (62) (Figure 1). P-selectin expression on platelet surface (which interacts with P-selectin granulocyte ligand 1, PSGL1, constitutively expressed by neutrophils) initiates platelet-neutrophil interaction (85, 86) and occurs after vessel injury/endothelial activation (87), pathogen recognition (88), or aging (89). P-selectin-dependent platelet-neutrophil interaction recruits downstream integrin-dependent pathways and culminates in neutrophil activation and facilitated extravasation (90). Neutrophils interacting with platelets can either: (a) phagocytose them, quenching their thrombogenic and inflammatory potential (85); (b) progressing to the generation of NETs (88), an event influenced by the neutrophil metabolic state (66, 91, 92).

Vital neutrophil internalization (emperilopolesis) into megakaryocytes occurs in the bone marrow. Engulfed neutrophils provide megakaryocytes with activating stimuli (causing a rise in platelet production) and donate membrane segments causing enhanced phosphatidylserine expression by chimeric daughter platelets (93). It is tempting to speculate that emperilopolesis holds the key to understanding the mechanisms of the later interactions between neutrophils and platelets in the circulation.

Platelets and megakaryocytes communicate long-range with neutrophils through exocytosed mediators and microparticles with enhancing actions on neutrophil activation (94, 95). The prototypic alarmin/damage-associated molecular pattern HMGB1, either as a soluble moiety or loaded into microparticles, is a potent promoter of extended neutrophil survival and of NET generation (40, 51, 96–99). Cooperation and bidirectional exchange of lipid metabolites between neutrophils and platelets through microparticles maximize the synthesis of prostaglandins, such as the pro-coagulant and vasoconstrictor signal, thromboxane A2 (100, 101) and to the activation of the complement cascade, which in turn promotes neutrophil recruitment and activation (102).

### An Anthology of Recurring *Topoi*: Shared Events Linked to Platelet and Neutrophil Biology

The interaction between platelets and neutrophils impacts on multiple stereotyped pathological manifestations (Table 1). A first hint can be found in laboratory tests, such as blood cell counts. In most cases, platelet and leukocyte numbers roughly correlate with systemic inflammation (147), either due to disease activity or infections. By contrast, reduced blood cell counts are a hallmark of a minority of autoimmune conditions, including SLE and overlap syndromes and Felty's syndrome, but might dominate during sepsis, affecting survival. No specific evidence is available about the reciprocal interactions between platelets and neutrophils during severe thrombocytopenia as an isolated phenomenon. This fact might also be due to difficulties in uncoupling variations in platelet counts and neutrophil responses from shared inciting stimuli underlying both events at an experimental level. Nonetheless, consistent evidence from sepsis, idiopathic thrombocytopenic purpura, thrombotic thrombocytopenic purpura and thrombocytopenia induced by heparin or other drugs suggests that neutrophils are intravascularly activated and tend to generate NETs in association with low platelet counts, possibly contributing to dysregulated hemostasis and adverse clinical outcomes (148–153). Interestingly, viral infections causing thrombocytopenia seem to associate with the expansion of low-density neutrophils (which are thought to have a higher propensity to form NETs), as observed in autoimmune diseases, such as SLE (154). Pancytopenia is a distinctive feature of hemophagocytic lymphohistiocytosis, a severe disorder developing either as a complication of multiple autoimmune diseases or as a standalone disorder (155). Variations in cell volume are thought to correlate with cytoskeletal remodeling and changes in cell function and metabolism (156–159). Mean platelet volume (MPV) is a variable provided in the context of blood count, significantly susceptible to the effects of multiple confounders at the analytical and preanalytical level. Altered MPV has been detected in SLE, RA, large- and small-vessel vasculitides, systemic sclerosis (SSc) and chronic spontaneous urticaria (103, 104, 117, 118, 123, 129, 135, 136, 160). However, disease activity in these settings has been associated with high and low MPV values, preventing a straightforward translation of this laboratory tool into clinical practice (161). Gasparyan et al. (137) and Scherlinger et al. (97) offer a possible syncretistic perspective: high platelet volume might reflect chronic low-grade platelet activation (156), whereas low MPV can be consequent to more severe inflammation, causing extensive shedding of platelet microparticles (32). Neutrophils showing increased cellular volume and consequent lower granular density have long been recognized as a hallmark of systemic immune-mediated diseases (159), although they are also detectable in sepsis (162) and cancer (163). This cell population is characterized by enhanced ability to extravasate, extended survival and by a tendency to form NETs (68, 158, 164). By contrast, consistent with the model of inverse association between NETosis and phagocytosis (Figure 1), low density neutrophils are less able to engulf substrates (159).

**Table 1:** Clinical and laboratory features potentially affected by altered platelet-leukocyte interactions in selected immune-mediated diseases.

	**Increased ischemic risk**	**Abnormal thrombin generation**	**Thrombotic microangiopathy**	**Lung involvement**	**Leukopenia**	**Thrombocytopenia**	**Altered platelet volume**	**Low complement**	**Vasculitis**	**Urticaria**	**Vascular remodeling**	**Fibrosis**	**References**
SLE	+	+	+/–	+	+	+	+	+	+/–	+/–	–	–	(34, 103–112)
APS	+	+	+/–	+	–	+/–	?	+/–	+/–	–	–	–	(113–116)
SSc	+	+/–	+/–	+	–	–	+	+/–	–	–	+	+	(117–122)
AAV	+	+	–	+	–	–	+	–	+	+/–	–	–	(123–128)
LVV	+	?	–	–	–	–	+	–	+	–	+	–	(126, 129–134)
RA	+	+	–	+/–	–	–	+	–	+/–	–	–	–	(119, 135–140)
CSU	–	+	–	–	–	–	+	+/–	+/–	+	–	–	(141–146)

*AAV, ANCA-associated vasculitides; APS, anti-phospholipid syndrome; CSU, chronic spontaneous urticaria; LVV, large-vessel vasculitides; RA, rheumatoid arthritis; SLE, systemic lupus erythematosus; SSc, systemic sclerosis. +, common association/typical feature; +/–, possible manifestation; –, infrequent manifestation; ?, lack of sufficient data*.

Patients with inflammatory diseases of the blood vessels (124, 130, 131), connective tissue diseases and chronic arthritides have higher risks of ischemic events (105, 106, 113, 135, 138, 139, 165–167). Accelerated atherosclerosis is a common finding in SLE (7, 106, 168–170), RA (7, 139, 165, 171), or systemic vasculitides (170). Inflammation-related atherosclerotic mechanisms are only partially understood (172). NETs are major determinants of endothelial dysfunction and have been detected in atherosclerotic lesions (173). Platelets are also involved in the early phases of atherosclerosis, where they facilitate leukocyte egress toward the subendothelial vascular layers (174, 175). Antiphospholipid antibodies (aPL) constitute a major independent risk factor in the antiphospholipid syndrome (APS), a condition characterized by arterial or venous thrombosis and/or pregnancy complications occurring as a standalone disorder or secondary to SLE and other autoimmune diseases (176). aPL promote HMGB1-related response in platelets and monocytes (177) and, in cooperation with platelet Toll-like receptor 4, induce NETosis (178), possibly contributing to immunothrombosis (76). In addition, they might impair microparticle scavenging by glycoprotein I, increasing the likelihood of pro-coagulant platelet-neutrophil activation (179). Besides atherosclerotic lesions, NETs have been detected into coronary thrombi (96, 180).

Aberrant coagulation is detectable in immune mediated diseases (34, 107, 108, 119). Altered coagulation cascade and increased cardiovascular risk are common in asthmatic patients (181, 182), while patients with chronic spontaneous urticaria show aberrant thrombin generation but suffer no excess prevalence of cardiovascular disease (125, 141–146).

Thrombotic microangiopathy, consisting in diffuse deposition of thrombi along small vessels due to widespread endothelial activation with hemolysis and platelet consumption, might complicate SLE, SSc, antiphospholipid syndrome and other immune-mediated diseases (183–185). Under intravascular hemolytic conditions, free hemoglobin favors platelet activation. In turn, activated platelet boost neutrophil activation, possibly further feeding the inflammatory cascade (186). In addition, sera from patients with thrombotic microangiopathies fail to degrade NETs, which, in turn, can trigger thrombosis (76, 187).

The lung is a major inflammatory target (188). Neutrophils and platelets undergo unique pathophysiological interaction with the lung vasculature, a reservoir for neutrophils that can thus easily respond to airborne infectious or sterile inflammatory stimuli (189). Unleashed neutrophil effector functions contribute to acute lung injury/acute respiratory distress syndrome (ARDS) during sepsis or vasculitis (50), to the long-term effects on bronchial tissue of chronic inflammation in cystic fibrosis (190, 191) and to interstitial fibrosis in SSc (40, 192). NET generation has a central role in neutrophilic lung injury (193, 194). The lung vasculature influences platelets since lung microvessels are (a) a site of maturation of platelet precursors from the bone marrow (20); (b) a niche for human and mouse megakaryocyte homing (23, 195); (c) a site of detoxification of platelet mediators, such as serotonin (196). Conversely platelets contribute to persistent vasoconstriction (potentially leading to pulmonary hypertension), interstitial fibrosis (197), and NET generation in acute lung injury (198).

## Abnormal Platelet-Neutrophil Interactions in Selected Disease Settings

### Systemic Vasculitides

Systemic vasculitides encompass a very large set of immune-mediated diseases characterized by vascular injury and downstream organ ischemia as the core pathophysiological event (199). They can be roughly classified into two main subsets, large- and small-vessel vasculitides, based on (prominent) sites of vascular involvement. From a pathophysiological point of view, vascular remodeling with immune infiltration, disassembly of the physiological extracellular architecture and aberrant proliferation of macrophages, fibroblasts and endothelial cells predominate in large-vessel vasculitides, resulting in stroke-like acute failure of large portions of tissues or entire organ due to vessel occlusion. By contrast, small-vessel vasculitis are characterized by necrosis associated or not to granulomatous inflammation and increased thrombotic risk (200). Altered cross-talk among platelet, neutrophils and microparticles might contribute to vascular injury (201).

Neutrophils cause vascular damage in small-vessel vasculitis, possibly reflected by the accumulation of leukocyte cellular debris in peri-vasculitic lesions (leukocytoclasia). In anti-neutrophil cytoplasmic antibody (ANCA)-associated vasculitides (AAV), neutrophil mediated vascular injury is part of a vicious circle linking exposure of myeloperoxidase (MPO) and/or proteinase-3 from granules to neutrophil surface or into the setting of NETs (202) to the development of ANCA, which in turn activate neutrophils and the vascular endothelium (3, 67). In patients with AAV neutrophil responses couple with platelet activation (126). Consistently, patients with active AAV show increased plasmatic levels of P-selectin (127) and HMGB1 (203, 204). Elevation of HMGB1 has also been detected in other vasculitic settings, such as IgA-vasculitis (formerly Henoch-Schonlein's purpura) and Kawasaki disease (205).

In large vessel-vasculitides, activation of platelets may contribute to promote vascular remodeling through the release of signals, such as VEGF (206). Platelets in large-vessel vasculitides show signs of activation (129, 201), and in giant cell arteritis are significantly increased in number during active disease (147) and form hetero-aggregates with leukocytes, possibly contributing to exacerbate ischemic risk (132, 201). This evidence provides a rationale for the use of aspirin in primary prevention (207) and for the employment of platelets as diagnostic surrogates (126). Results in the literature (208) and reports on a potential cyclooxygenase-independent mechanism for aspirin in giant cell arteritis (209) suggest however that the role of platelets should be interpreted with caution.

Little is known on platelet-leukocyte interactions in large vessel vasculitides, although relative depletion of neutrophil granule content has been reported in giant cell arteritis in association with platelet activation (126, 132). Notably, items of small-vessel (peri)vasculitis have been reported in large series of temporal artery biopsies from patients with giant cell arteritis (210) and might be secondary to stereotyped events resembling those observed in small-vessel vasculitides, possibly targeting the *vasa vasorum* and entailing aberrant platelet-neutrophil cross-talk.

### Systemic Lupus Erythematosus

SLE is a multi-organ autoimmune disease with a wide spectrum of clinical manifestations and pathogenic mechanisms (211). Failure of clearance mechanisms and/or exposure of cell death debris in an inflammatory setting promotes autoimmunity and subsequent tissue damage (212). Hematological manifestations constitute a hallmark of SLE and are detected in >80% of patients (213). Cytopenia is the most frequent modality of presentation and affects either red blood cells, platelets and leukocytes. Bone marrow abnormalities are frequent, although no clear correlation can be established with disease activity (214–217). Accordingly, primary bone marrow failure is a rare cause of cytopenia (218), with most relevant mechanisms (besides drugs) being inflammation-induced iron deficiency and cytolysis. Neutropenia occurs in up to one third of SLE cases, in most cases due to antibodies (219), which is not apparently associated to infectious risk (109). Thrombocytopenia is also common in patients with SLE (220). Megakaryocyte number is generally increased during disease activity, reflecting extensive platelet production (214, 220). Patients with SLE and thrombocytopenia have an increased risk of a severe disease course and of mortality in large cohort studies (221).

Cardiovascular manifestations are frequent in patients with SLE and a cause of morbidity and mortality (222). Accelerated atherosclerosis, aPL and dysfunctional coagulation likely converge to determine this risk (106). Despite low absolute platelet counts, patients with SLE frequently show extensive platelet activation (223–227). Higher levels of P-selectin are detectable in urines from patients with lupus nephritis (228). Platelets also contribute to mesangial remodeling and renal vascular damage (229, 230).

Endothelial derived microparticles constitute the most abundant microparticle subset in patients with SLE and correlate with endothelial dysfunction and interferon-α signature (231, 232). However, PDμP also accumulate during active SLE (233, 234) and might impact on inflammation and hemostasis (34, 234). PDμP facilitate coagulation by providing phosphatidylserine scaffolds and intravascularly expressed TF. In addition, they promote neutrophil activation and NET generation being reservoirs of HMGB1 (96) and CD40L (234). Finally PDμP synergise with NETs as inducers of anti-nuclear immunity by constituting a source of mitochondria, which behave as potent damage-associated molecular patterns due to their bacterial origin (235).

Mechanistically, platelet activation in SLE might depend on circulating immunocomplexes, which are abundant in SLE patients biological fluids and are recognized on platelet surface by FcγRIIA and Toll-like receptor 4,7 (236). PDμP themselves could take part in immunocomplexes, enforcing an inflammatory/immunogenic self-sustaining loop (235). Ensuing complement activation in turn amplifies and propagates neutrophil and platelet activation (102, 231, 234).

### Systemic Sclerosis

SSc is a systemic autoimmune disease, characterized by unrelenting inflammation with a wound repair response consisting in mesenchymal extracellular matrix deposition leading to fibrosis, and by microvascular dysfunction and aberrant neoangiogenesis (120, 237, 238). Platelets and aberrant platelet-neutrophil interactions play a role in SSc (239). Possibly in response to microvascular damage, platelets of patients with SSc are constitutively activated and express signals driving neutrophil interaction (240, 241). P-selectin dependent cell-cell interactions seem to be relatively less represented in SSc, due to the lower leukocyte expression of PSGL-1 (242). Neutrophils have a pericellular distribution of granules and of their content, causing enhanced degradation of fibrinogen by exposed neutrophil proteases and eventually impairing fibrinogen dependent interactions between neutrophil CD11b/CD18 (also known as Mac-1 or αMβ2 integrin) and platelet glycoprotein IIbIIIa (40, 96). Indeed, platelet-neutrophil heterotypic aggregates are less frequently detected in SSc compared to other inflammatory conditions (40, 96, 242).

Activated platelets in SSc contribute to impaired vascular tone [due to altered arachidonic acid metabolism (197, 239)] and to fibrosis. In fact, platelets release multiple fibrogenic mediators, such as transforming growth factor beta, platelet-derived growth factor, CXCL4 (also known as platelet factor 4), beta-thromboglobulin, serotonin and HMGB1 (27, 97, 242–244). NETs promote fibroblast differentiation and function and might synergise with platelet in supporting fibrosis (192), also in light of the abundance of NET byproducts in the blood of patients with SSc (40). Synergistic NET/platelet-induced fibrosis is expected to be particularly significant in lung tissue, where neutrophil and platelets are abundant (189). Consistently, PDμP (retrieved from the plasma of patients with SSc) induce neutrophil granule mobilization and autophagy, culminating in extended neutrophil survival and generation of NETs through a HMGB1-dependent mechanism. Furthermore, neutrophils stimulated by SSc platelet microparticles migrate in murine lungs, associate with interstitial endothelial damage and promote lung fibrosis (40).

### Rheumatoid Arthritis

Rheumatoid arthritis is a relatively frequent autoimmune disease characterized by prominent involvement of the synovial joints. Although extra-skeletal manifestations are relatively less frequent compared to other immune-mediated diseases, patients with RA show an increased ischemic risk, pointing to the existence of a core pathophysiological event linking inflammatory manifestations to vascular dysfunction (245).

Neutrophils undergoing NET generation might provide autoantigens in RA. Patients in fact frequently develop antibodies against citrullinated peptides (ACPA). Citrullination occurs thanks to the activity of deiminating enzymes, such as protein-arginine deiminase 4 (PAD4), abundantly expressed in neutrophils (246). Citrullinated histones constitute a fundamental component of chromatin threads within NETs (73, 247). Platelets might also contribute to ACPA formation due to their expression of vimentin, a preferential target of citrullination (248). ACPA sustain joint inflammation by perpetuating macrophage activation within the synovia, eventually causing chronically elevated levels of tumor necrosis factor alpha (TNFα), which in turn promotes synovial proliferation, bone reabsorption, neoangiogenesis and enhanced synovial infiltration through activated endothelium (249). Enhanced expression of TF on activated platelets and neutrophils, which coexist in the synovial fluid of inflamed joints in patients with RA, provides an interesting hint on potential mechanisms involved in RA-associated enhanced ischemic risk. Platelets and leukocytes from patients with RA are activated due increased plasmatic concentration of TNFα (140). Indeed, platelet respond to TNFα thanks to the expression of TNF receptors 1 and 2 (250) resulting in increased P-selectin expression, platelet degranulation, phosphatidylserine up-regulation and TF expression. TNFα-activated platelets prompt thrombin generation and activation of leukocytes due to P-selectin. Consistently, platelet and leukocyte activation is reduced in patients treated with anti-TNF agents (140), who also face relatively lower rates of cardiovascular events in the long-term (251, 252).

Platelets can be activated by collagen through the megakaryocyte lineage-specific glycoprotein VI and thus prompted to generate microparticles. Boilard and colleagues (253) showed that, following this mechanism, high concentrations of PDμP (possibly shuttled by engaging leukocytes) are detectable in synovial fluid of patients with RA and are required for arthritis development in a murine model. PDμP contain significant amounts of interleukin 1α and β, promoting synoviocyte proliferation, and of IL8, enhancing neutrophil recruitment and ensuring maintenance of inflammation (253).

## Conclusion

Platelets and neutrophils are major determinants of the immune-hemostatic continuum and extensively interact based on cell-cell contact and/or exchange of soluble signals and microparticles to synergise in contrasting the noxious effects of endogenous or environmental stimuli toward vessel and tissue integrity and to promote physiological tissue renewal and homeostasis. These events, part of a set of simple, innate, but evolutionarily preserved stereotyped responses, are disproportionately active and self-sustained in patients with immune-mediated diseases, such as systemic vasculitides, SLE, SSc, RA and possibly allergic disorders and might account for the development of either some inflammatory manifestations and of cardiovascular complications. Patients with immune-mediated diseases consistently show signs of platelet (and/or PDμP) activation, possibly prompting either the formation of heterotypic aggregates with neutrophils (as in giant cell arteritis) or neutrophil activation toward enhanced survival and eventually NET generation (as observed in small-vessel vasculitis, SLE, RA and to a higher extent SSc). Diagnostic and therapeutic strategies currently employed in the setting of autoimmune diseases to prevent disease progression and the occurrence of secondary complications are generally not targeted on these pathogenic mechanisms, suggesting the existence of a largely unexplored window of opportunity to improve survival and quality of life for patients by dampening sustained neutrophil-platelet interactions.

## Author Contributions

GR and NM collected the literature data, drafted, and revised the manuscript. AM revised the manuscript and provided critical analysis of intellectual content. All authors approved the final version of the manuscript and agree to be accountable for all aspects of the work in ensuring that questions related to the accuracy or integrity of any part of the work will appropriately be investigated and resolved.

### Conflict of Interest

The authors declare that the research was conducted in the absence of any commercial or financial relationships that could be construed as a potential conflict of interest.
